# Children with Obesity Experience Different Age-Related Changes in Plantar Pressure Distributions: A Follow-Up Study in China

**DOI:** 10.3390/ijerph17186602

**Published:** 2020-09-10

**Authors:** Yihong Zhao, Debin Zheng, Shiyang Yan, Mengyuan Liu, Luming Yang

**Affiliations:** 1 National Engineering Research Center of Clean Technology in Leather Industry, Sichuan University, Chengdu 610065, China; zhaoyihong@stu.scu.edu.cn (Y.Z.); zhengdebin@stu.scu.edu.cn (D.Z.); shiyangy@kth.se (S.Y.); 2 Key Laboratory of Leather Chemistry and Engineering, Ministry of Education, Sichuan University, Chengdu 610065, China; liumengyuan@stu.scu.edu.cn

**Keywords:** obesity, children, age, loading transference, plantar pressure redistribution, follow-up study

## Abstract

Age is a key factor in plantar pressure distributions during the development of obese children. However, the existing evidence for age-related plantar pressures of obese children is not sufficient to make clear how the plantar pressures would change with the increasing age. This study aimed to evaluate the plantar pressure redistributions of obese children after a three-year follow-up and to further compare these changes with normal-weighted children. Ten obese children and eleven normal-weighted counterparts were involved in this study. Plantar pressure measurements were undertaken using a Footscan^®^ plantar pressure plate on two test sessions three years apart. Peak pressure, pressure-time integral, standard maximum force, and z-scores of these variables were analyzed. Loading transference analyses were applied to detect the different loading transferring mechanisms between obese and normal-weighted children. Significantly increased plantar pressures were observed at the lateral forefoot and midfoot for obese children, which gradually deviated from those of normal-weighted children over the 3 years. With the increasing age, obese children displayed a lateral loading shift at the forefoot in contrast to the normal-weighted. Early interventions are cautiously recommended for obese children before the plantar loading deviation gets worse as they grow older.

## 1. Introduction

Altered gait patterns and plantar pressures caused by obesity have been a closely followed issue due to its relationship with balance and a higher risk of injuries [1,2,3,4,5]. Excess weight could lead to specific alterations in plantar pressures, especially for children whose musculoskeletal system is immature and developing [6]. Inconsistencies exist, however, in the effects on plantar pressure caused by obesity, when it comes to different age ranges [7,8,9,10]. Specifically, the plantar pressure of obese children is evidently higher than that of children with normal weight at the midfoot at the age of 4, whereas at the midfoot and forefoot at 8 years old [10]. Obese children, aged 8–11 years old, generate higher peak pressures at the lateral forefoot, midfoot, and lateral heel compared with the normal-weighted children [9]. But the distinctions at the heel are not found when the children are at younger ages ranged from 5 to 10 years [8]. Besides, with the increasing age, the greatest maximum force tends to shift towards more lateral plantar regions in obese children than in normal-weighted ones [7,8]. Taking these discrepancies into consideration, age is likely to play a diverse role in the development of plantar pressure for children with obesity, compared to normal-weighted children.

While most previous reports have focused on the development of plantar pressure distributions with age in normal-weighted children [11,12,13,14], studies on age-related effects on plantar pressure in obese children are still rare. To the best of our knowledge, so far, only Steffen et al. [15], based on a cross-sectional study, found that the midfoot loading of obese children showed a 1.48-fold increase at one-year-old compared to the normal-weighted, and the value increased to 3.49-fold at the age of 10. Despite this existing work, the age-related plantar pressure distributions remain poorly recognized. Given that the constant biomechanical stress could lead to long-term implications during growth and development, it is imperative to ascertain the effects of age on the plantar pressures in obese children and the age-related differences compared with their normal-weighted counterparts.

In addition, individual unique exists in children [13,16], especially for the obese population, since individuals could develop different altered foot loading patterns due to obesity. On the other hand, the individual changes could be further amplified during the development with the increasing age. Follow-up research was thus proposed in this study to provide insights into the longitudinal evolution in the development of plantar pressure for obese children and to avoid the potential inaccuracy caused by individual discrepancies in cross-sectional studies.

In doing so, the present study aimed to discuss the age-related plantar pressure redistributions in obese children and to explore the specific plantar regions subject to increased plantar pressures. This was done by comparing the redistributions after a three-year follow-up between obese and normal-weighted children. Further, age-related loading transferences were compared between obese and normal-weighted children to provide some support for the differences in plantar pressure redistributions with the increasing age.

## 2. Materials and Methods

### 2.1. Participants

Seventy children aged 8 years old at the baseline were recruited in a randomly selected primary school in Yantai city, China. The baseline and follow-up experiments were conducted on two separate occasions approximately three years apart. To avoid the potential confounding effects of foot deformity and typical gaits on the plantar pressures [17,18], all the participants were screened by means of an interview and physical examination for abnormal foot or gait, e.g., hallux valgus, flat foot, high-arch foot, toe-out, and toe-in gaits. Participants were categorized as normal-weighted, overweight, and obese by the recommendations of body mass index (BMI) adjusted for age and gender [19]. A study group of ten participants who maintained obese over the follow-up study were selected for further investigation. A control group was formed by eleven participants who stuck to normal weight over this period. The basic information of the participants is shown in Table 1.

Written informed consent was obtained from all the participants and their legal guardians prior to the test.

### 2.2. Experimental Procedures

A one-meter Footscan^®^ plantar pressure plate (RSscan International, Paal, Belgium) was used to collect foot loading parameters with a sampling rate of 250 Hz. The plate was mounted in the middle of a five-meter long rubber walkway at the same height. After a familiarization of the “two-step” initial protocol [20], children were asked to walk through the plate barefoot at their self-comfort speed. Trials were considered satisfactory when both feet made full contact with the plate. At least three valid experiments were required for each participant. Data of the habitual foot (all were the right one in this study) of each subject were taken for further analysis [21].

### 2.3. Data Processing

Ten plantar regions were defined by Footscan^®^ software (RSscan International, Paal, Belgium) and artificial adjustment pixel by pixel according to the anatomical principal: hallux (T1), the second to fifth toes (T2–5), the first to fifth metatarsals (M1–M5), midfoot (MF), heel medial (HM), and heel lateral (HL). The following plantar pressure variables were evaluated: peak pressure (PP, kPa), pressure-time integral (PTI, kPa·s), and standard maximum force normalized to the body weight (SMaxF, %). The SMaxF was calculated as follows (Equation (1)):

SMaxF = MaxF/(Weight × *g*) × 100% (1)

Z-score, as a useful mathematical tool for monitoring developmental deviation in obese children [22], was calculated for those plantar pressure variables of obese children in this study. It was based upon the data of the control group at the baseline and the follow-up, respectively. Z-scores was calculated as follows (Equation (2)):

z-score = (Po − P*_N_*)/SD*_N_* (2)

where P*_o_* represented the pressure parameters (PP, PTI, and SMaxF) of obese children, P*_N_* represented the mean values of the pressure parameters (PP, PTI, and SMaxF) of normal-weighted children, and SD*_N_* represented the standard deviation of the pressure parameters (PP, PTI, and SMaxF) of normal-weighted children.

Further, in order to interpret the age-related loading transferences, force-time integral normalized to the total impulse (FTI*rel*; %) was involved, and the regional ΔFTI*rel* was then expressed as a difference value of FTI*rel* between the baseline and follow-up. ΔFTI*rel* was calculated according to the following formulas (Equations (3) and (4)):

FTI*rel* = FTI/∑FTI × 100% (3)

ΔFTI*rel* = FTI*rel_B_* − FTI*rel_F_* (4)

where *FTIrel_B_* represented the regional FTI*rel* in children at the baseline; *FTIrel_F_* represented the regional FTI*rel* in children at the follow-up.

An algorithm, based on anatomical foot structures and roll-over gait of children, was adopted to quantitatively assess the age-related loading transferences [23]. Accordingly, the foot plantar was divided into four levels: the toe regions for level 1 (L1), the forefoot regions for level 2 (L2), the midfoot region for level 3 (L3), and the heel for level 4 (L4). Then, the load shift in the lateral-medial direction was evaluated within levels. Finally, the posterior-anterior loading transferences along the longitudinal arch were assessed between levels. Of note, the load would transfer from the area with a positive value of ΔFTI*rel* to the area with a negative one until a 0 is reached. Besides, the load transfers between the adjacent areas in preference to others.

### 2.4. Statistical Analysis

All data were proved to be normally distributed by one-sample Kolmogorov–Smirnov test (*p* > 0.05). Subsequently, independent-samples *t*-tests were performed to evaluate differences in plantar pressures (PP, PTI, and SMaxF) between the baseline and follow-up for both of the two body types. Confidence intervals (CI) at 95% and effect sizes (ES) by Cohen’s d were calculated for all the significant mean differences. Effect sizes were reported as trivial (0–0.2), small (0.2–0.6), moderate (0.6–1.2), large (1.2–2), and very large (2–4) [24]. All statistical analyses were performed by SPSS version 22 (IBM, Armonk, NY, USA).

## 3. Results

### 3.1. Age-Related Changes in Plantar Pressure Distributions

Results of PP, PTI, and SMaxF for obese and normal-weighted children with significant differences, 95% confidence intervals, and effect sizes are shown in Table 2.

Peak pressures of both groups rose significantly in all regions after the follow-up check, and obese children showed a greater increase than the normal. With respect to the baseline data, both the obese and the normal displayed higher peak pressures at M2–M4 and the heel. After three years, however, with the increase of age, the main loading areas shifted to M4 and M5 in obese children but to the heel in normal-weighted children. Pressure-time integrals of obese children increased significantly in all plantar regions after the follow-up. While no significant differences were found at T1, M3, M4, and MF in the control group. Additionally, for the obese children, the follow-up values of pressure-time integrals were over two times higher at M3–M5 than the baseline. Obese children generated higher peak pressures and pressure-time integrals than normal-weighted children in all regions, both at the baseline and follow-up. But the case was largely altered when the maximum force was normalized to the body mass. Compared to the normal-weighted children, the obese showed an upward trend for SMaxF at M2–M5 and a downward trend in the rest areas, both at the baseline and follow-up. With respect to the age-related impact, the hallux and midfoot demonstrated a decrease in SMaxF in obese children over this period. Of note, SMaxF values at M3–M5 increased during this follow-up, and the significance was found at M5 (*p* = 0.024, ES = 1.19), whereas the opposite trend was observed in normal-weighted children.

### 3.2. Age-Related Plantar Pressure Deviations in Obese Children

**Figure 1:**
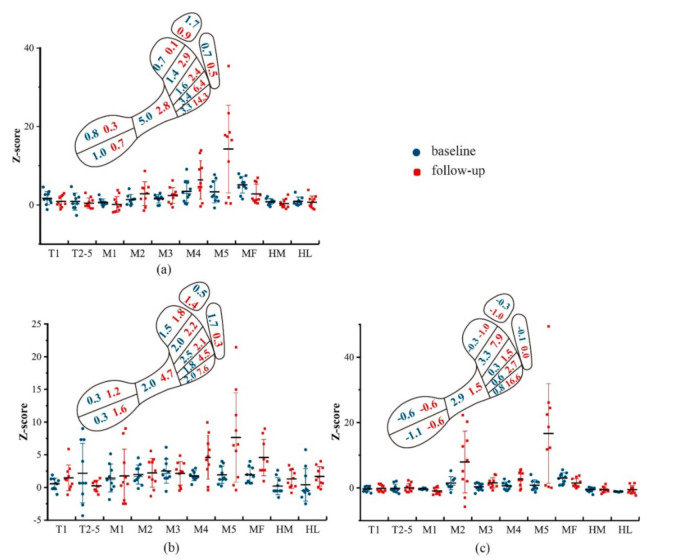
Mean values of z-scores of plantar pressures ((**a**) peak pressure; (**b**) pressure-time integral; (**c**) standard maximum force) for obese children at baseline and follow-up. Blue numbers in the footprint represented the mean value of z-scores at baseline, and red numbers in the footprint represented the mean value of z-scores at baseline.

Z-scores of the pressure variables for obese children at the baseline and the follow-up are provided in Figure 1. It was evident that the z-scores of those variables in obese children went higher at M4 and M5 with the development of the age. At the midfoot, obese children displayed increased z-scores of PP and SMaxF but a decreased z-score of PTI at the follow-up.

### 3.3. Age-Related Loading Transferences in Obese Children

**Figure 2:**
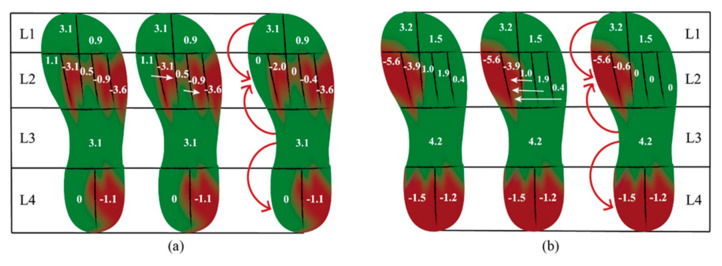
Age-related loading transferences in (**a**) obese children and (**b**) normal-weighted children.

The assessment of load transference was performed based on the values of ΔFTI*rel* and is illustrated in Figure 2. After a three-year development in obese children, M2, M4, and M5 were the main areas to which the load transferred, in the lateral-medial direction. The control group, however, shifted the impulse more medially within the forefoot level (from M3–M5 to M1 and M2). In the posterior-anterior direction, plantar loadings were shifted from the toes and midfoot to the forefoot and heel in the present follow-up, for both of the obese and control groups.

## 4. Discussion

It is vital for obese children to figure out the aging influences on plantar pressure distributions. In this study, we evaluated the age-related alteration of plantar pressure distribution in obese children, and in the meantime, we explored the deviation of plantar pressures from normal-weighted children over the session. The results indicated that plantar pressures of obese children redistributed in a different way from normal-weighted children during the follow-up session, with a more lateral load shift and increasing deviation.

As agreed with the previous work [12,25], peak pressures at all plantar regions of normal-weighted children statistically increased with the developmental age. In this study, obese children also generated significantly greater peak pressure across all plantar regions after the follow-up, with the effect sizes from moderate to very large (ranged from 1.42 to 2.42), and it was more remarkable at M3–M5. This age-related difference between the obese and the normal-weighted at the lateral forefoot was also identified by the data of pressure-time integral. When compared with the baseline, values of pressure-time integrals at those areas of obese children were over two times higher, while no evident changes were found in normal-weighted children. As a result, the lateral forefoot turned out to be the main loading areas during this growing process, as opposed to the most heel loading in the normal-weighted population. This finding was partly in accordance with the previous studies that have confirmed elevated plantar loadings in obese children at the lateral forefoot in comparison with the normal-weighted ones [7,26,27]. Apart from this, a greater increase of peak pressure and pressure-time integral with the increasing age was found at the lateral forefoot in obese children in our study. This could be caused by some age-obesity-related adjustments, of which two seemed to be the most likely. Firstly, children with obesity have been confirmed to exhibit an increased ankle external rotation in the early stance phase as well as a greater knee internal rotation in the late stance phase [28]. This adaptation leads to a more external foot progression angle, pressures thus orient laterally at the forefoot, especially in the late stance during walking [29]. It has to be mentioned that this was analyzed in relation to children’s lower-extremity musculoskeletal system, which would make a constant adjustment with the development. This could, in return, aggravate the differences in plantar pressure distribution at the lateral forefoot between obese and normal-weighted children overtime. Secondly, as obese children gained more weight than their normal-weighted counterparts during this period, the increasing body mass would call for greater joint moments as well as more efforts for propulsion, contributing to the age-related aggravation in plantar loading at the lateral forefoot.

Given the potentially confounding effects of body mass, we developed the standard maximum force as well as the loading transference assessment to have an insight into the age-related plantar pressure redistributions. Differences in plantar pressure distribution between obese and normal-weighted children changed dramatically once normalized to their individual weight. However, the higher loadings at the central and lateral forefoot (M2–M5) and midfoot remained in children with obesity, which was in total agreement with the finding of Cousins et al. [26]. These findings supported the view that the lateral forefoot and midfoot could be the most vulnerable sites developing higher plantar pressures. Bosch et al. [30] divided the foot into five regions (hindfoot, midfoot, forefoot, hallux, and toes) and found standard maximum forces at the whole forefoot increased with age in normal-weighted children. In the present study, however, normal-weighted children demonstrated significantly increased standard maximum forces at M1 and M2 and decreased values at the lateral forefoot with the developmental age. A possible reason for such inconsistency is that the more detailed masking of plantar regions in the present study helps to distinguish where and how the forces were altered with age at the forefoot more preciously. Interestingly, despite the absence of statistical significance, obese children tended to undertake higher standard maximum force at the lateral forefoot with aging, which was in contrast to the normal-weighted ones. In this way, our results supported the opinion of Butterworth et al. [31] that obesity would increase the plantar loadings not only via increased body weight but also via biomechanical alterations of lower limb structures. With respect to the loading transference derived from the difference of relative force-time integrals, obese children displayed a lateral load shift at the forefoot with aging, while the controls transferred more medially. Such difference reaffirmed that the underlying influences caused by obesity on the biomechanics of plantar loading could accumulate over time, thus aggravating the pressure differences at the lateral forefoot in comparison with the normal-weighted cohort.

In an attempt to figure out whether the increasing age could enlarge the plantar pressure difference between obese and normal-weighted children, deviation of plantar pressure distribution from the normal-weighted children was further quantified by z-score, a statistical tool for growing deviation assessment [22,32]. Notably, at the midfoot, the increasing age narrowed the deviation of peak pressure and standard maximum force but enlarged the deviation of pressure-time integrals. This could be interpreted with the age-related changes of those variables at the midfoot. Specifically, no significant differences of peak pressure as well as a standard maximum force were found at the midfoot between follow-up and baseline for both the obese and control groups, and the same held true for pressure-time integrals in normal-weighted children. But remarkably elevated PTI at the midfoot was observed in the obese group. Consequently, although obese children managed to prevent higher deviations of peak pressure and standard maximum force, it was still insufficient to compensate for the constantly increasing pressure-time integrals, causing higher repetitive plantar loads overtime at the midfoot. Obese children are subject to a flatter foot, with a potential trend of collapse in the medial longitudinal arch [15,33]. In this regard, it is conjectured that the foot arch of obese children failed to disperse the foot loadings as appropriately as in the normal-weighted children, leading to continuously increasing repetitive impulses at the midfoot in obese children from 8-year-old to 11-year-old.

### Limitations of the Study

There are two main limitations to this present study that should be taken into account. Firstly, although the Footscan^®^ plantar pressure plate has been shown to be a reliable and valid tool for plantar pressure measurements [34,35], they are limited to the forces perpendicular to the surface. A combination with forces in other planes, such as the shear forces, could better evaluate the impacts of age on foot loadings. Secondly, our study solely involved the natural walking movement, and it is suggested to concern running and other movement modes in the future relative research to draw a more comprehensive conclusion. Finally, it is important to note that the children’s age was limited to 8–11 years old in our analysis, and further studies are needed to determine whether the results are applicable to other age groups among children.

## 5. Conclusions

The results of this study confirmed the age-related plantar pressure redistributions in obese children, with a lateral loading transference observed at the forefoot. Moreover, aggravated plantar deviation from the normal-weighted children was identified with the increasing age. Evident increases and deviations related to age at the lateral forefoot and the midfoot were found for most pressure variables of obese children. Additionally, the magnitude of effect size for significant differences between the follow-up and baseline ranged from 0.96 to 3.73, which are perceived to be meaningful in clinical application. These findings identified the accumulated adverse effects of childhood obesity with increasing age and provided an insight into the age-related plantar pressure redistributions in obese children, suggesting that lateral forefoot and midfoot were the key areas concerning the age-related continuous increase in plantar pressures over the 3-year period. Accordingly, we cautiously suggest the early intervention in childhood obesity in relation to their developmental age to correct for potential deviations and prevent the disproportional increase of plantar pressures.

## Figures and Tables

**Table 1:** Basic information of the participants.

	Baseline	Follow-Up	*p*	Mean Difference	95% Confidence Interval
**Obese Group**					
Number (*n*)	10	10			
Age (years)	8	11			
Height (cm)	136.3 ± 4	160.1 ± 5.8	0	23.8	−28.5 to −19.1
Body mass (kg)	43.4 ± 5.4	70.3 ± 6	0	26.9	−32.3 to −21.5
BMI (kg/m^2^)	23.3 ± 2.2	27.5 ± 2.2	0	4.14	−6.2 to −2.1
**Control Group**					
Number (*n*)	11	11			
Age (years)	8	11			
Height (cm)	126.9 ± 4.7	149.7 ± 5.8	0	22.82	−27.5 to 18.1
Body mass (kg)	25.7 ± 3.7	39.1 ± 6.9	0	13.37	−18.4 to −8.5
BMI (kg/m^2^)	15.9 ± 1.3	17.3 ± 2.2	0.081	1.44	−3.1 to 0.2

**Table 2:** Mean (SD) values for plantar pressures with MD (95% CI) and effect sizes for significant difference between follow-up and baseline.

Follow-Up vs. Baseline in Obese Group				Follow-Up vs. Baseline in Control Group		
	Follow-Up	Baseline	MD (95% CI), ES	Follow-Up	Baseline	MD (95% CI), ES
**PP (kPa)**						
T1	126.7 (48.3) ^a^	57.4 (20.3)	69.3 (34.5 to 104.1), 1.87	96.7 (33.7) ^b^	37.4 (11.8)	59.3 (34.3 to 84.4), 2.40
T2–5	54.7 (24.6) ^a^	13.5 (6.5)	41.1 (23.3 to 59.1), 2.42	45.6 (19.2) ^b^	10.9 (3.2)	34.7 (21 to 48.3), 2.40
M1	88.3 (36.2) ^a^	41.2 (10.6)	47.1 (20.6 to 73.5), 1.81	85.8 (17.7) ^b^	33.3 (12.1)	52.5 (38.7 to 66.2), 3.49
M2	214.6 (91.2) ^a^	91.1 (22.2)	123.5 (52.6 to 194.4), 1.86	129.3 (30.5) ^b^	67.8 (16.6)	61.5 (39.4 to 83.7), 2.55
M3	230.1 (98.8) ^a^	102.7 (17)	127.4 (56.3 to 198.5), 1.84	112.6 (49) ^b^	77.6 (15.6)	35 (2.6 to 67.3), 0.96
M4	264.7 (137.2) ^a^	94.2 (28.4)	170.5 (71.4 to 269.5), 1.72	85.7 (28.2) ^b^	60.4 (9.9)	25.3 (6.5 to 44.1), 1.20
M5	351.2 (223.4) ^a^	49.4 (19.6)	301.8 (141.7 to 461.8), 1.90	66.1 (19.8) ^b^	25.6 (6.9)	40.6 (27.4 to 53.8), 2.73
MF	68.6 (29.8) ^a^	37.5 (8.5)	31.1 (9.4 to 52.8), 1.42	35.1 (12.4) ^b^	16.7(4.4)	18.4 (9.7 to 27.1), 2.01
HM	191 (71.5) ^a^	84.9 (12.1)	106.1 (54.7 to 157.6), 2.07	169.7 (63.6) ^b^	71.5 (15.8)	98.2 (57 to 139.4), 2.12
HL	176.6 (63.2) ^a^	78.7 (11.3)	97.8 (52.3 to 143.4), 2.21	147.4 (41.4) ^b^	67.9 (11.3)	7.9 (51.1 to 107.8), 2.62
**PTI (kPa·s)**						
T1	28.3 (13.7) ^a^	17.9 (7.3)	10.4 (0.9 to 20.7), 0.95	18 (7.4)	13.6 (7.9)	4.4 (−2.7 to 11.6)
T2–5	8.5 (3.6) ^a^	2.9 (2.7)	4.6(1.5 to 7.7), 1.43	7.4 (4.4) ^b^	2.6 (0.6)	4.8 (1.8 to 7.7), 1.61
M1	30 (16.5) ^a^	14.6 (4.1)	15.4 (3.4 to 27.3), 1.31	22.9 (4.4) ^b^	11.7 (1.7)	11.2 (8 to 14.4), 3.29
M2	60 (24.2) ^a^	34.6 (8.8)	25.4 (8.3 to 42.5), 1.39	35.7 (11.1) ^b^	22.9 (5.8)	12.8 (4.4 to 21.1), 1.40
M3	61.7 (25.3) ^a^	38.3 (7.3)	23.5 (5 to 41.8), 1.26	31.8 (14.1)	28.4 (3.5)	3.4 (−6.8 to 13.5)
M4	75.8 (36.5) ^a^	32 (4.4)	43.8 (17.6 to 70), 1.73	25.6 (11.4)	21.3 (5.8)	4.2 (−4.2 to 12.7)
M5	78.3 (54.6) ^a^	15.6 (5.2)	62.7 (23.6 to 101.8), 1.62	17.4 (8.1) ^b^	8 (3.6)	9.4 (3.7 to 15.1), 1.50
MF	20.7 (8.5) ^a^	11.7 (3.6)	9 (2.3 to 15.7), 1.36	7.1 (3)	6 (3.2)	1.1 (−1.7 to 3.8)
HM	44 (12.8) ^a^	23.5 (7.8)	20.5 (10.6 to 30.5), 1.94	32.6 (9.3) ^b^	22.2 (5.6)	10.4 (3.5 to 17.3), 1.36
HL	42.4 (12) ^a^	22.4 (10.2)	20 (9.6 to 30), 1.81	29.1 (8.5) ^b^	20.9 (4.2)	8.2 (2.2 to 14.1), 1.22
**SMaxF (%)**						
T1	17 (7.5)	18.6 (6.7)	−1.6 (−8.3 to 5.1)	18.4 (7.3)	21.3 (8.3)	−2.8 (−10 to 4.3)
T2–5	7.4 (5.2)	7.2 (4.3)	0.2 (−4.2 to 4.7)	7.2 (4.1)	7.6 (3.4)	−0.4 (−3.9 to 3.1)
M1	20.2 (9.1)	15.8 (1.7)	4.5 (−2.1 to 11)	29.8 (9.6) ^b^	18 (7.4)	11.8 (4.2 to 19.4), 1.74
M2	40.2 (19.8) ^a^	20.1 (4.3)	20.1 (5.7 to 34.5), 1.48	23.6 (2.1) ^b^	17.2 (0.9)	6.5 (4.5 to 8.6), 3.73
M3	28.8 (12.4)	21.1 (4.7)	7.6 (−1.2 to 16.4)	16.8 (7.8)	19.8 (4.1)	−3 (−8.6 to 2.5)
M4	26.4 (12.1)	17.9 (4.2)	8.5 (−0.5 to 17.4)	11.9 (5.3)	15.7 (3.5)	−3.8 (−7.8 to 0.2)
M5	29 (21.4) ^a^	10.6 (4.8)	18.4 (2.9 to 33.8), 1.19	5.7 (1.4)	8.1 (3)	−2.4 (−4.7 to 0)
MF	28.8 (13.3)	33.6 (9.3)	−4.7 (−15.5 to 6)	14.6 (9.6)	17.2 (5.6)	−2.5 (−9.8 to 4.8)
HM	39.6 (14.6)	31.6 (5.2)	8 (−2.8 to 18.7)	49 (16.4) ^b^	35.7 (7.2)	13.3 (1.7 to 24.9), 1.05
HL	34.1 (13) ^a^	24 (0.6)	10.1 (0.9 to 19.4), 1.28	39.9 (10.1) ^b^	30 (5.3)	9.9 (2.6 to 17.3), 1.23

MD represents the mean difference between plantar pressure values at follow-up and baseline. ^a^ Represents a significant difference between follow-up and baseline for obese children (*p* < 0.05). ^b^ Represents a significant difference between follow-up and baseline for normal-weighted children (*p* < 0.05).
